# Chemical Modifications of Nucleic Acid Aptamers for Therapeutic Purposes

**DOI:** 10.3390/ijms18081683

**Published:** 2017-08-02

**Authors:** Shuaijian Ni, Houzong Yao, Lili Wang, Jun Lu, Feng Jiang, Aiping Lu, Ge Zhang

**Affiliations:** 1 Institute of Precision Medicine and Innovative Drug Discovery, School of Chinese Medicine, Hong Kong Baptist University (HKBU), Hong Kong 999077, China; jack6shuai@163.com (S.N.); yaohouzong@163.com (H.Y.); wanglili9413@163.com (L.W.); ljaaa111@163.com (J.L.); 2 Institute for Advancing Translational Medicine in Bone & Joint Diseases, School of Chinese Medicine, Hong Kong Baptist University (HKBU), Hong Kong 999077, China; 3 Faculty of Materials Science and Chemical Engineering, the State Key Laboratory Base of Novel Functional Materials and Preparation Science, Ningbo University, Ningbo 315211, Zhejiang, China

**Keywords:** nucleic acid aptamer, nuclease degradation, rapid excretion, binding affinity, chemical modification

## Abstract

Nucleic acid aptamers have minimal immunogenicity, high chemical synthesis production, low cost and high chemical stability when compared with antibodies. However, the susceptibility to nuclease degradation, rapid excretion through renal filtration and insufficient binding affinity hindered their development as drug candidates for therapeutic applications. In this review, we will discuss methods to conquer these challenges and highlight recent developments of chemical modifications and technological advances that may enable early aptamers to be translated into clinical therapeutics.

## 1. Introduction

In 1990, several groups isolated the first nucleic acid aptamers by “SELEX” (Systematic Evolution of Ligands by Exponential Enrichment) or “in vitro selection” (a demarcation resulting from whether the technique was learned from Tuerk and Gold [1] or Ellington and Szostak [2], respectively). Through 3D conformational complementarities, aptamers bind to a wide range of targets, including small metal ions and organic molecules, peptides, proteins, viruses, bacteria, whole cells and even targets within live animals [3]. Being similar to the binding of antibodies and antigens, the binding between aptamer and its target has comparable binding affinity and specificity, which makes aptamers a promising class of therapeutic alternatives to antibodies [4].

In addition, nucleic acid aptamers have minimal immunogenicity, high chemical synthesis production, low cost and high chemical stability, drawing extensive attention of researchers to the development of aptamer therapeutics [5].

**Figure 1:**
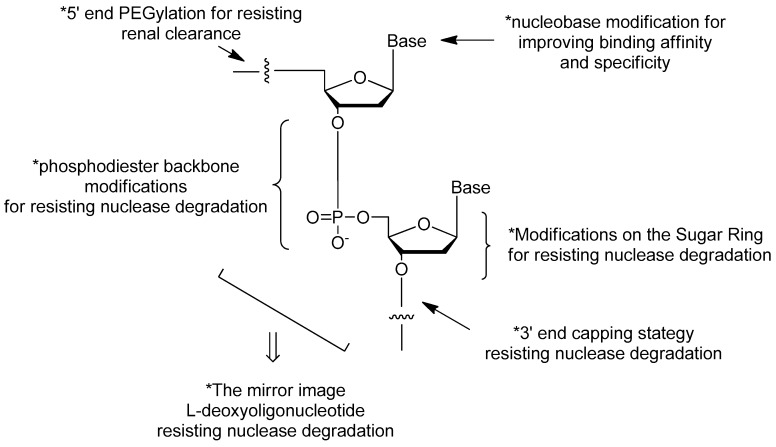
The common strategies in the chemical modifications of nucleic acid aptamers and their purposes. Among the modifications, such as modifications on the terminals of nucleic acids, modifications on the phosphodiester linkage, modifications on the sugar ring and modifications on the bases, the 3′ end capping with inverted thymidine [6,12] and PEGylation [13] have been the common strategies in the chemical modifications of nucleic acid aptamers for development clinical therapeutics [14,15,16,17].

However, the susceptibility to nuclease degradation and rapid excretion through renal filtration severely limit the practical usage of aptamers [6,7]. Many aptamers with potent activities have unacceptable short half-lives in vivo [8,9]. Besides, the binding affinity and specificity of unmodified nucleic acid aptamers are sometimes insufficient for successful implementation as therapeutic agent [10]. The generation of high quality aptamers from conventional SELEX is generally below 30% [11]. Therefore, many attempts of post-SELEX chemical modifications should be done in order to solve these challenges (Figure 1).

In this review, the standard synthetic method of solid phase phosphoramidite chemistry for nucleic acid aptamers preparation will be introduced firstly [18,19]. Then, the chemical modification strategies of aptamers for resisting nuclease degradation [12,20,21,22,23,24,25,26], improving target binding affinities [10,27,28,29,30,31] and resisting renal clearance [32,33,34,35,36] will be summarized, sequentially. Among the modifications, such as modifications on the terminals of nucleic acids, modifications on the phosphodiester linkage, modifications on the sugar ring and modifications on the bases, the 3′ end capping with inverted thymidine [6,12] and PEGylation [13] have been the common strategies in the chemical modifications of nucleic acid aptamers for development clinical therapeutics (e.g., pegaptanib [14,15,16,17], etc.). More excitingly, aptamers with improved binding affinities are being generated with modifications on the bases [29] or substitutions of two non-bridging phosphate oxygen atoms in nucleic acids by sulfur replacement [10] (see “SOMAmers” and “PS2 walk” below).

## 2. Chemical Synthesis of Nucleic Acid Aptamers

### 2.1. Synthesis of DNA Aptamers

DNA aptamers can be synthesized through the classic solid phase phosphoramidite four-step process on the automated DNA synthesizer [18]. 

**Figure 2:**
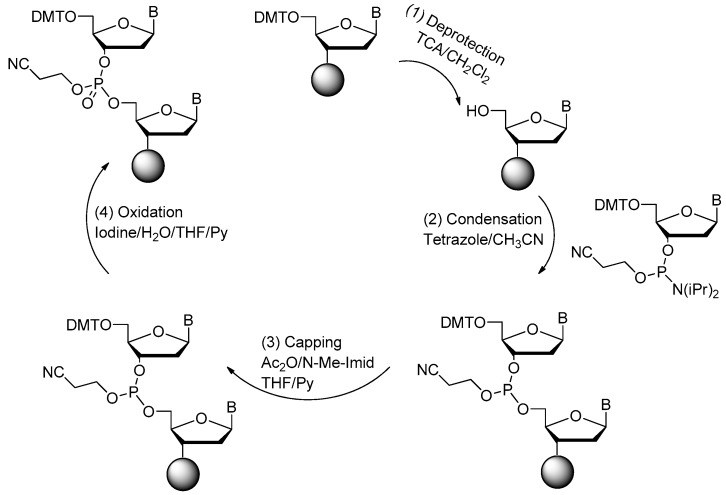
Four-step phosphoramidite oligodeoxynucleotide synthesis cycle (adapted from [18]). The phosphoramidite method, pioneered by Marvin Caruthers in the early 1980s, and enhanced by the application of solid-phase technology and automation, is now firmly established as the method of choice. Phosphoramidite oligonucleotide synthesis proceeds in the 3′ to 5′ direction (opposite to the 5′ to 3′ direction of DNA biosynthesis in DNA replication). One nucleotide is added per synthesis cycle. The phosphoramidite DNA synthesis cycle consists of a series of steps outlined in the figure.

The four-step method is shown in Figure 2. First, the 4,4′-Dimethoxytriphenylmethyl (DMT) group is removed from the deoxynucleoside (5′-end) which is linked to the control pore glass (CPG) columns. Large excess of acid solution (trichloroacetic acid (TCA)) could be used for the deprotection of DMT. In the second step of the cycle, an internucleotide bond called phosphite trimester is synthesized. Then, in the third step, the reaction product from Step 2 should be treated with capping agent to cap the unreacted free 5′-OH group. In the last step (Step 4), the new phosphite is oxidized to the corresponding phosphotriester by iodine. The cycle is repeated, once for each base, to produce the required oligonucleotide. Finally, the nucleic acid aptamers could be cleaved from the CPG by concentrated ammonium hydroxide. The protecting groups for phosphates and heterocyclic bases could be removed at the same time [18,37,38].

At present, the application of four-step method is very common. For the most part, progress in the solid phase nucleic acid synthesis field has not changed this fundamental approach. For R&D purposes, shortened aptamers with 20 to 50 nucleotides in length can be generated in individual labs using “lab scale” DNA or RNA synthesizers [39] (e.g., Expedite 8909, ABI394). 

### 2.2. Synthesis of RNA Aptamers

**Figure 3:**
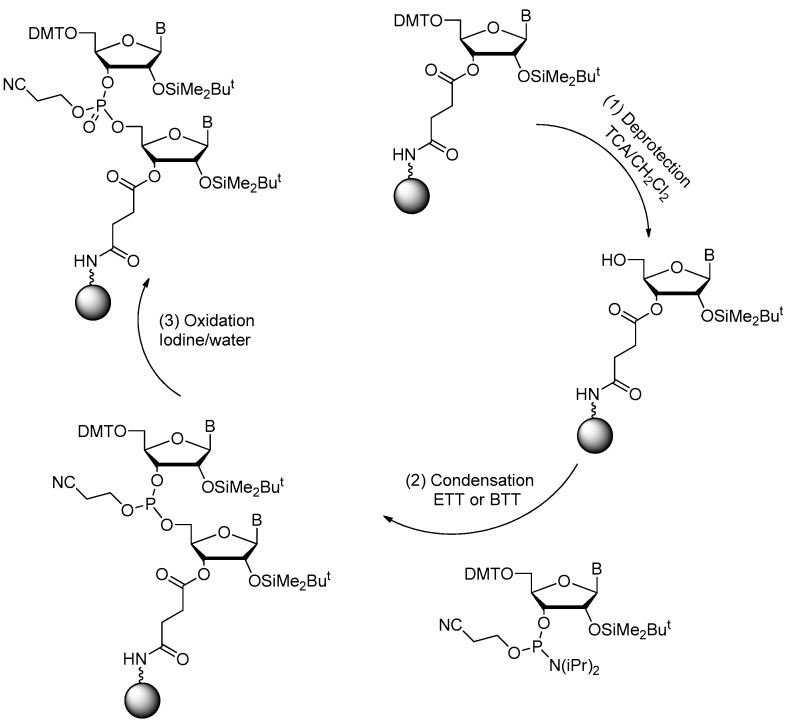
Solid-phase RNA synthesis via the phosphoramidite method (adapted from [19]). In RNA synthesis, the 2′-hydroxy group is protected with TBDMS (*t*-butyldimethylsilyl) group, which can be removed by treatment with fluoride ion.

Several of synthetic strategies for the solid-phase synthesis of RNA had been reported [19,40,41,42]. Among the combinations of different coupling/activation chemistries and protecting groups for the 2′-hydroxyl and exocyclic amine groups, the tert-butyldimethylsilyl protection of the ribose 2′-hydroxyl group combined with the standard protecting groups for the exocyclic amine groups (benzoyl for adenosine, acetyl for cytidine, and isobutyryl for guanosine) were most widely used [40,43]. Phosphoramidite monomers were usually activated with 4,5-dicyanoimidazole, 5-ethylthio-1*H*-tetrazole (ETT) or 5-benzylthio-1*H*-tetrazole (BTT) (Figure 3). The solid supports for RNA synthesis were polymeric supports or CPGs with different linkers and pore sizes. The final product could be cleaved from the CPG by concentrated ammonium hydroxide. The protection groups can also be removed at the same time [19,40,44,45,46].

## 3. Modifications of Nucleic Acid Aptamers

### 3.1. Aptamer Derivatives for Resisting Nuclease Degradation

#### 3.1.1. Terminal 3′–3′ and 5′–5′ Internucleotide Linkage

**Figure 4:**
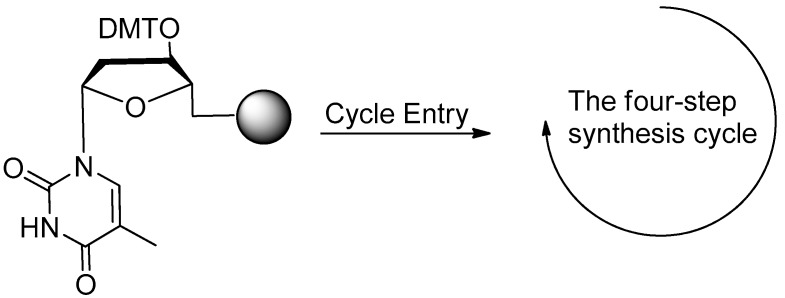
Solid-phase synthesis of 3′-inverted dT modified aptamers. Synthesis of 3′-inverted dT modified aptamers needs modified CPG with the 5′-hydroxyl of the first nucleoside attached, followed by chain elongation in standard 3′→5′ fashion.

The 3′–3′ and 5′–5′ inversions were tested in 1991 by Seliger et al. [12]. The 3′-end capping with inverted thymidine has also been a common strategy among aptamers for diseases therapy in ongoing or completed clinical trials [15,47]. Research suggested that 3′-inverted dT modification could increase the stability and resistance of aptamers to 3′-exonuclease in human serum. Synthesis of 3′-inverted dT modified aptamers (Figure 4) needed modified CPG with the 5′-hydroxyl of the first nucleoside attached, followed by chain elongation in standard 3′→5′ fashion [12,20,21].

#### 3.1.2. 3′-Biotin Conjugates

**Figure 5:**
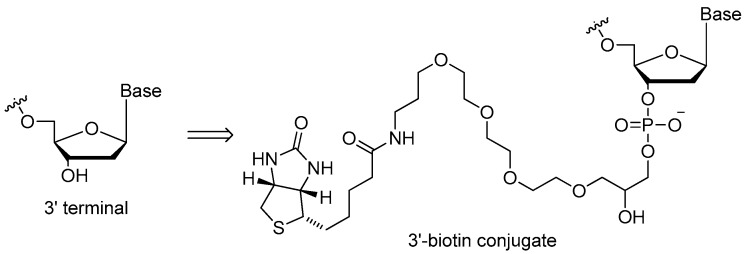
Structure of the 3′-biotin conjugate. 3′-Biotin could inhibit the activity of 3′-exonuclease, which was similar to 3′-inverted dT modification. In addition, the 3′-biotin conjugates slowed down the clearance rate in blood circulation system in vivo.

In some ways, 3′-biotin (Figure 5) could resist the activity of 3′-exonuclease, which was similar to 3′-inverted dT modification. Dougan et al. [36] investigated the 3′-biotin-streptavidin conjugates of the thrombin aptamer to find that the 3′-biotin rendered resistance to the 3′-exonuclease in the blood of mouse or rabbits. In addition, the 3′-biotin-streptavidin conjugates slowed down the clearance rate of aptamers in blood circulation system in vivo [36]. A similar 3′-biotin approach was also used to protect the DNA aptamer targeting the SARS coronavirus helicase for up to 31 and 16 h in 5% and 10% fetal bovine serum, whereas the original aptamer can only sustain half of that time [20].

#### 3.1.3. Modifications on the Sugar Ring

##### 2′-Substitutions

**Figure 6:**
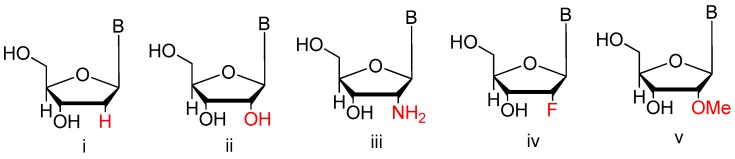
2′-substitutions utilized to enhance the stability of aptamers in vivo (adapted from [39]). 2′-Substitutions can easily be incorporated into aptamers during chemical synthesis and include: (**i**) 2′-H; (**ii**) 2′-OH; (**iii**) 2′-NH_2_; (**iv**) 2′-F; and (**v**) 2′-OMe.

Modifications to the sugars such as 2′-fluoro (2′-F) or 2′-amino (2′-NH_2_) ribose groups (Figure 6) on the pyrimidine residues have been available for incorporation into enzymatically derived nucleic acids for some years. Although both are effective at improving serum half-life, 2′-F modifications quickly garnered favor over 2′-NH_2_ due to the increased coupling efficiency during solid-phase synthesis, and elimination of extra deprotection steps during 2′-NH_2_ purification. The more bulky 2′-O-methyl (2′-OMe) modifications have been previously used as a post-selection modification due to their increased nuclease resistance and high duplex melting temperature which could be seen in the clinical examples [48,49].

##### LNA, UNA, 2′-F ANA

**Figure 7:**
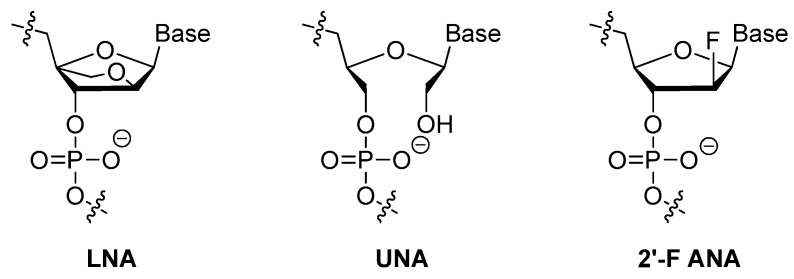
Structures of Locked nucleic acid (LNA), unlocked nucleic acid (UNA) and 2′-deoxy-2′-fluoro-d-arabinonucleic acid (2′-F ANA). LNA is an analog of ribonucleotide with a methylene linkage between 2′-O and 4′-C of the sugar ring. UNA misses a bond between C2′ and C3′ of the sugar ring. 2′-F ANA adopts anti-conformation with 2′-F-G.

Locked nucleic acid (LNA) (Figure 7) is an analog of ribonucleotide with a methylene linkage between 2′-O and 4′-C of the sugar ring. This modification showed great resistance to nucleases and increased thermostability thus could be used to generate the most stable pairs [50,51]. Darfeuille et al. also found that the LNA/DNA chimera LNA5, a stable complex that against HIV-1 trans-activating response (TAR) RNA, was able to maintain the intact structure within 20 h in bovine serum [52]. Shi et al. developed a new LNA/DNA chimeric aptamer probe through proper LNA incorporation and 3′-3′-thymidine (3′-3′-T) capping. The serum stability of original aptamer was gradually enhanced while its specificity and affinity were perfectly maintained. Especially TD05.6 aptamer which had a 7-base pair-LNA substitution exhibited a ten-fold elevated stability in serum and a much slower clearance rate in mice [53].

Unlike LNA, a structurally rigid modification that increases the thermostability of a modified-oligonucleotide thus protects it from nucleases degradation in cells, unlocked nucleic acid (UNA) (Figure 7) in which a bond between C2′ and C3′ of the sugar ring was absent makes aptamers more flexible [54]. Due to its nature of flexibility, UNA could alleviate strain in tight loop structures. Pasternak et al. found that UNA modifications on the loop regions of a 15-mer thrombin targeted DNA aptamer increased its thermodynamic stability. However, modifications within the G-quartet structures were unfavorable for quadruplex formation [55]. They also demonstrated that UNA could be placed in many positions without affecting the thrombin-binding affinity and anticoagulant efficiency of the aptamer [55].

It has been found that modifications at the 2′-position of the sugar ring would bring about different effects on thermostability based on the molecularity of G-quadruplex. Peng et al. discovered that, in both anti-HIV phosphorothioate aptamer and thrombin-binding aptamer, substitution of guanines (G) that adopted anti-conformation with 2′-F-G could maintain the quadruplex conformation, while substituting guanines with syn-conformation was not favored [25]. More importantly, two 2′-F-modified thrombin-binding aptamers (PG13 and PG14) showed approximately four-fold increased binding affinity to thrombin and up to seven-fold higher nuclease resistance. As a result, the 2′-deoxy-2′-fluoro-d-arabinonucleic acid (2′-F ANA) (Figure 7) modification was very suitable for improving the biological and physicochemical properties of DNA G-quartets [25].

#### 3.1.4. Modifications on the Phosphodiester Linkage

##### Methylphosphonate or Phosphorothioate

**Figure 8:**
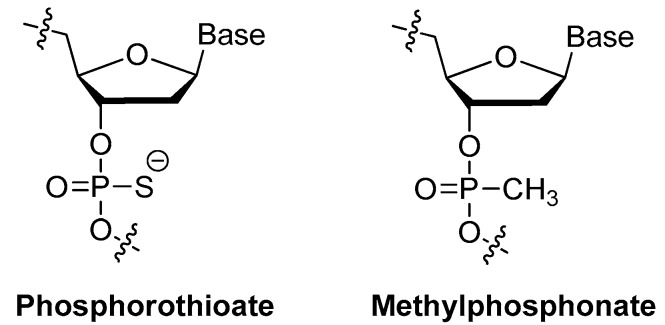
Structures of methylphosphonate and phosphorothioate.

Replaced phosphodiester linkage of DNA with methylphosphonate or phosphorothioate analog is commonly used for aptamer modification. Thermodynamic studies revealed that loss of the negative charge of the phosphate backbone, as the methylphosphonate analog (Figure 8), destabilized the G-quadruplex structure [56]. The ionic radii of the oligonucleotide backbone atoms also have an impact in the stabilization of G-quadruplex structures. Sacca et al. found that substitution of the phosphate backbone atom O with S (phosphorothioate analog, Figure 8) might influence the thermal stability of the G-quadruplex structure in a molecularity-dependent manner [56].

The thermodynamic stability of the phosphodiester linkage of the thrombin-binding aptamer d(GGTTGGTGTGGTTGG) with thiophosphoryl substitutions at different internucleotide sites were studied [23,24]. Complete substitution by thiophosphorylated oligonucleotides was limited as their high toxicities, so partial substitutions with the maximum thermal stability were selected for evaluating their stabilities under conditions of nuclease RQ1 DNAse hydrolysis and their antithrombin activities in blood plasma [24]. Aptamer d(GGSTSTSGGTGTGGSTSTSGG) with thio-substitutions in both TT loops exhibited similar antithrombin efficiency to the unmodified aptamer but better resistance to the degradation of DNA nuclease in blood serum [23].

More recently, phosphorodiothioate linkages (PS2) were employed to stabilize phosphate backbone. The substitution of both non-bridging oxygen atoms with sulfur could give rise to a phosphorodithioate linkage, which, similar to natural DNA, is achiral at phosphorus. In addition, it was reported that PS2 substitutions dramatically improved target binding affinity by ~1000-fold (see PS2 walk below) [10].

##### Replaced by Triazole

**Figure 9:**
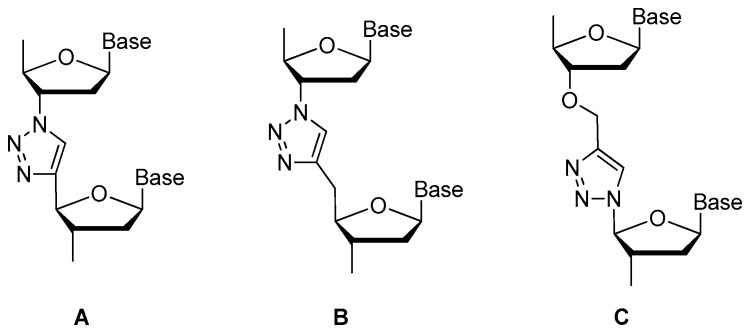
Fragments of oligonucleotide analogs with different types of triazole internucleotide modifications (adapted from [64]). A, B, C represent three different types of triazole internucleotide modifications.

Replacement of the oligonucleotide phosphodiester linkage with triazole linkages has shown great promise [57,58,59,60]. These triazole analogs can be obtained through automated phosphoramidite synthesis with modified dinucleoside blocks [61] or the click reaction between azide- and alkyne-bearing nucleosides [62,63]. Figure 9 shows three types of promising triazole internucleotide modifications [64].

**Figure 10:**
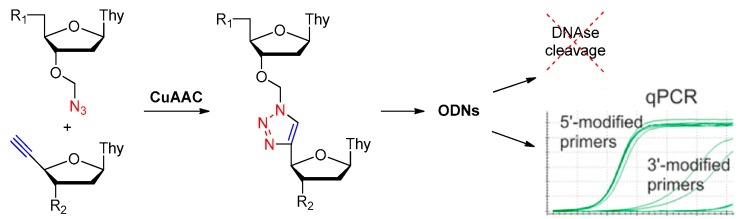
Synthesis of the triazole internucleoside linked oligonucleotide analogs with increased resistance to DNAses and polymerases (adapted from [64]).

Varizhuk et al. synthesized several new oligonucleotide analogs with triazole internucleotide linkages through the click reaction as shown in Figure 10. These analogs bore DNA hybridization affinities similar to those of original oligonucleotides and increased resistance to nuclease cleavage [64].

Later in 2013, Varizhuk et al. synthesized a series of triazole-modified DNA aptamers with structure similar to thrombin-inhibiting G-quadruplexes TBA15 (Thrombin-Binding Aptamer) and TBA31, then tested their secondary structure stabilities, binding affinities for thrombin and anticoagulant effects [65]. A modification in the central loop of the aptamer quadruplex resulted in an anticoagulant activity similar to that of TBA15. Although the modification failed to enhance thrombin binding affinity, it protected aptamers from nuclease hydrolysis thus increased their stabilities. The novel aptamers were potent thrombin inhibitors and could be an alternative to the known anticoagulant drugs [59].

#### 3.1.5. The Mirror Image l-DNA

**Figure 11:**
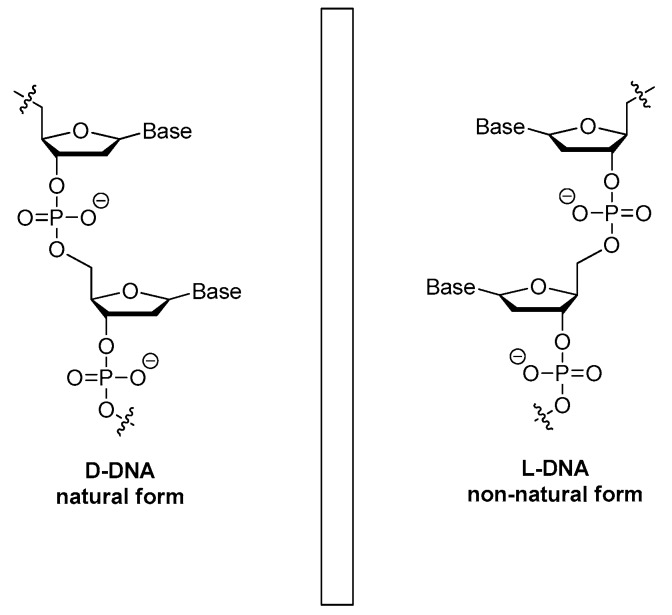
Structures of l-deoxyoligonucleotide (l-DNA). Mirror image aptamers are composed of non-natural l-ribose nucleotides. The molecules are initially selected from natural d-ribose aptamer libraries against a non-natural target, for example a d-peptide. Once optimized as a d-aptamer, the mirror image l-aptamer (Spiegelmer) is synthesized chemically and intrinsically bound to the natural l-target, such as a naturally occurring protein.

Natural DNAs are all in d-form. A chiral transition could result in the mirror image l-DNA (Figure 11) that may display high resistance to the degradation of nucleases and retain the affinity to targets. Based on the sequences of d-form aptamers, the l-enantiomeric oligonucleotide aptamers (also called as Spiegelmers) were then chemically synthesized [66]. Based on the domain approach, Purschke et al. found a 65-mer Spiegelmer that bound to a stable 25-amino acids length domain of bacterial staphylococcal enterotoxin B [64]. The l-DNA Spiegelmer showed comparable binding affinity to the l-peptide domain and slightly reduced affinity to the whole bacterial staphylococcal enterotoxin B protein.

Through an in vitro-selection process, which was started from a random pool of oligonucleotides, a 67-mer Spiegelmer with a dissociation constant (Kd) of 20 nM for gonadotropin-releasing hormone (GnRH) was reported by Wlotzka et al. [67]. This Spiegelmer was an effective antagonist to GnRH in Chinese hamster and castrated rat models. Besides, the PEGylated Spiegelmer showed more pronounced inhibition activity and longer plasma half-life [67]. Towards the same target, other Spiegelmers with high specificity and affinity were identified through the usage of Spiegelmer technology by Leva et al. [68]. Firstly, aptamers that bind to d-GnRH with Kd of 50–100 nM were isolated, and then their enantiomers were synthesized. The resulting Spiegelmers had similar affinities to that of d-aptamers [68]. Many clinical evaluated aptamers such as NOX-A12, NOX-H94 and NOX-E36 are all l-aptamers [69,70].

A number of different strategies and chemical modifications are now available to enhance the stability of aptamers to nuclease (Table 1). Among these modifications, 2′-fluoro or 2′-*O*-methyl-substitutions and 3′ end capping with inverted thymidine have been the common strategies in the chemical modifications of nucleic acid aptamers for resisting nuclease degradation.

### 3.2. Aptamer Derivatives for Resisting Renal Clearance

#### 3.2.1. 5′-End with Cholesterol

Even with stabilizing backbone modification, small aptamers are subjected to rapid excretion through renal clearance mainly through glomerular filtration. Formulation with bulky moiety enlarges the size of aptamers, overcoming the renal filtration and extending circulation time, evidently [32,33].

**Figure 12:**
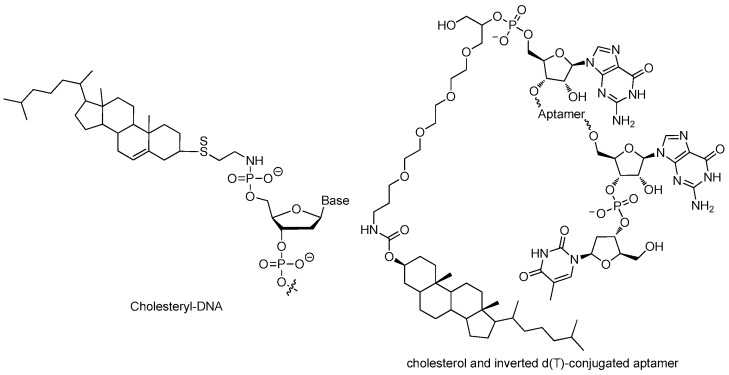
Structures of cholesterol-oligonucleotide conjugates (adapted from [71]). Cholesterol can be derivatized to the 5′-end of an aptamer to form a cholesterol-oligonucleotide (cholODN) conjugate. The half-time of the resulting cholODN in plasma was considerably longer than the control ODN.

Cholesterol can be derivatized to the 5′-end of an aptamer to form a cholesterol-oligonucleotide (cholODN) conjugate. Smidt et al. added cholesterol at the 5′-end of a 16-mer oligonucleotide (ODN) through a phosphate spacer (Figure 12), the half-time of the resulting cholODN (9–11 min) in plasma was considerably longer than the unmodified ODN (<1 min) [71]. The resulting cholODN can be further linked with low-density lipoprotein (LDL) to form cholODN-LDL complex that turned out to be stable against degradation by rat serum nucleases. The cholODN had a roughly 10-fold longer plasma half-life than the unmodified ODN [71].

Lee and coworkers modified a 29 nucleotide-long 2′-F pyrimidine modified RNA aptamer with cholesterol to form a cholesterol-conjugated aptamer (chol-aptamer) (Figure 12) which can be efficiently absorbed into the cell and inhibits Hepatitis C virus RNA replication [71]. The chol-aptamer had no toxicity in vitro or in vivo. It did not induce any notable alteration in the gene expression profile, including innate immune-related genes. Moreover, administration of the chol-aptamer was well tolerated in mice without any abnormalities observed. Noticeably, cholesterol conjugation showed longer half-life with approximately nine times lower of clearance rate in plasma. In other words, it extended the duration time that the aptamer stayed in plasma, thus enhanced the stability when the aptamer was exposed to body [32].

#### 3.2.2. 5′-End with Dialkyl Lipids

**Figure 13:**
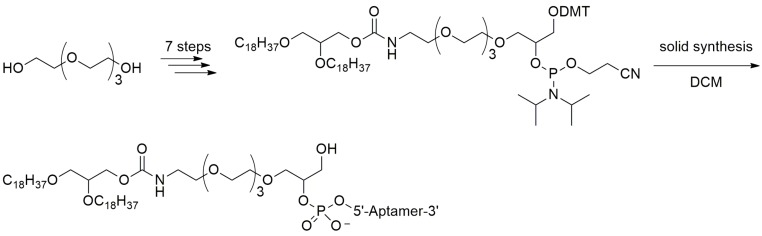
Synthesis of the dialkylglycerol (DAG) modified VEGF aptamer (adapted from [72]). Liposome-anchored aptamer maintained the high binding affinity to VEGF. Moreover, the plasma residence time was considerably improved when compared with that of the original aptamer.

Willis et al. reported the preparation and functional properties of a nuclease-resistant vascular endothelial growth factor (VEGF) aptamer which was attached to liposome bilayers through a lipid group. The resulting liposome-anchored aptamer maintained the high binding affinity to VEGF. Moreover, the residence time in plasma was considerably improved when compared with that of the original aptamer [72]. They used the solid phase phosphoramidite method to prepare a dialkylglycerol (DAG) modified VEGF aptamer in which two 18-carbon saturated unbranched hydrocarbon chains were attached via a tetraethylene glycol linker. The DAG phosphoramidite was synthesized in seven steps and then introduced to the 5′-end of the VEGF aptamer (Figure 13) [73]. Afterwards, the DAG-modified VEGF aptamer was incorporated into the bilayers of liposomes, which resulted in aptamers with improved inhibitory activity toward VEGF-induced endothelial cell proliferation in vitro and increased vascular permeability in vivo [73].

#### 3.2.3. 5′-End PEGylation

**Figure 14:**
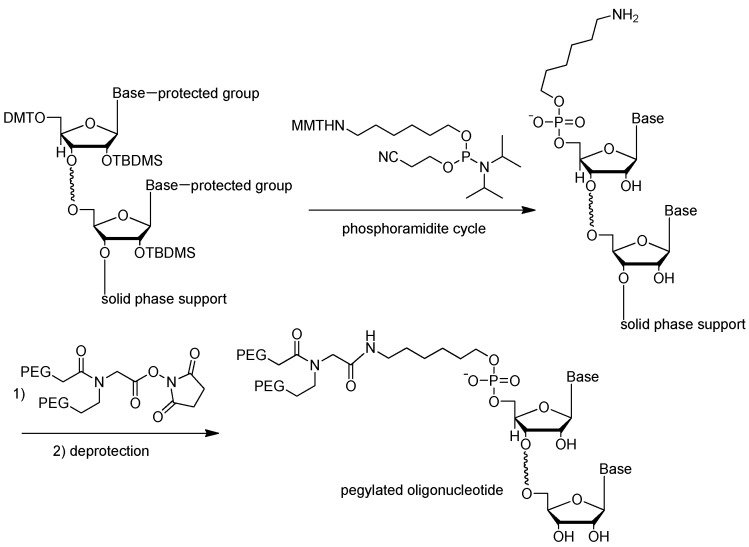
Addition of the aminolinker to 5′-end of the oligonucleotide and PEGylation of amino-modified oligonucleotide with 40 kDa Y-shaped PEG (*n* = ~450) (adapted from [74]). Amino-modified oligonucleotide could be reacted with NHS-ester-activated PEG to form oligonucleotide-PEG conjugate. Conjugation of aptamers with high molecular weight PEG could limit the rate of filtration and extended half-life up to 24–48 h.

In 2011, Hoffmann et al. described the PEGylation of amino-modified NOX-E36 oligonucleotide by using *N*-hydroxysuccinimide (NHS)-ester-activated polyethylene glycol (PEG), which was most widely used, especially for manufacturing large quantities of PEGylated oligonucleotides. Following synthesis and two-step deprotection, the resulting intermediate amino-modified oligonucleotide reacted with NHS-ester-activated PEG to form oligonucleotide-PEG conjugate (Figure 14). Other coupling methods such as activation by *p*-nitrophenyl carbonate or thiol-maleimide coupling could also be used [74]. The choice of coupling strategies should be made under consideration of the following factors: (1) compatibility with the oligonucleotide; (2) accessibility of the modified oligonucleotide; and (3) reactivity of the activated PEG, which should only react at the functionalization site of the oligonucleotide.

**Figure 15:**
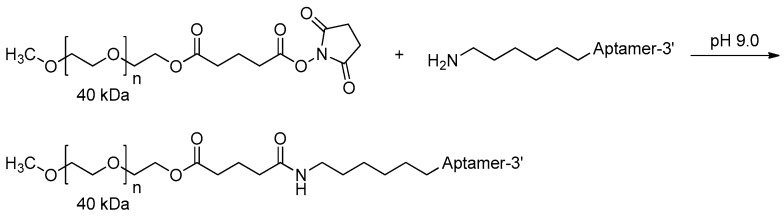
Reaction scheme of aptamer conjugating to a 40 kDa polyethylene glycol (PEG) at the 5′-termini (adapted from [76]).

MP7 is one of the DNA aptamers that bind specifically to the murine extracellular domain of PD-1 (Programmed death protein 1) and block the PD-1:PD-L1 (Programmed death-ligand 1) interaction. However, the unmodified DNA aptamer exhibited very short in vivo half-time (<1 h) owing to the rapid renal filtration of such small molecule [75]. It has been reported that conjugation of aptamers with high molecular weight PEG could limit the rate of filtration and extended half-life up to 24–48 h [32,75]. Thus, MP7 was modified at its 5′-termini with a 40 kDa PEG (Figure 15). The PEGylated form of MP7 retained the ability to block PD-1 binding to PD-L1, and significantly suppressed the growth of PD-L1 positive colon carcinoma in vivo [76,77] (Table 2).

### 3.3. Aptamer Derivatives for Improving Binding Affinity and Target Selectivity

#### 3.3.1. Modifications on the Bases; SOMAmers

**Figure 16:**
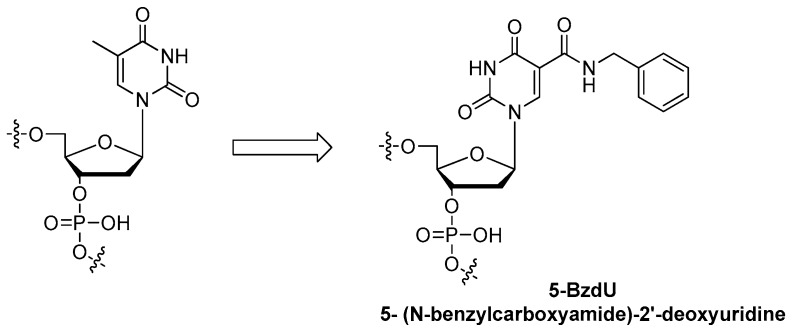
Structure of 5-BzdU (5-(*N*-benzylcarboxyamide)-2′-deoxyuridine).

Aptamers with improved binding affinities are being generated with modifications on the base. AS1411 aptamer is a 26-mer single strand DNA 5-d(GGTGGTGGTGGTTGTGGTGGTGGTGG)-3′ which binds to the nucleolin protein expressed on the surfaces of cancer cells [78,79,80]. Recent research has shown that 5-BzdU (5-(*N*-benzylcarboxyamide)-2-deoxyuridine) modification (Figure 16) of the AS1411 aptamer might selectively increase its targeting affinity to cancer cells while the normal healthy cells have no significant influence [26].

**Figure 17:**
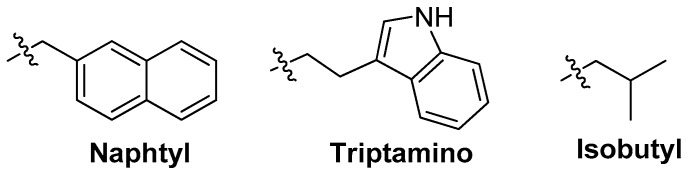
Structures of naphtyl, triptamino and isobutyl.

The benzyl could be replaced by the other functional groups such as naphtyl, triptamino, isobutyl and so on (Figure 17). These additional groups might increase the affinities of aptamers to their targets [81,82,83,84] (Table 3).

The base modifications have also made significant advancements to give aptamers protein-like functionality [11,87]. The SOMAmers (Slow Off-rate Modified Aptamers) not only display improved binding affinities and binding kinetics (in particular, slow off-rates) when compared to traditional aptamers, but also the inclusion of these modifications in their libraries significantly increased the selection “hit rate” [88]. The power of this kind of base modifications has been further demonstrated through the discovery of a 32 nucleotide SOMAmer, SL1025, which binds IL-6 with 200 pmol/L binding affinity and exhibits very little nuclease degradation over a 48-hour incubation in human serum [47,89].

#### 3.3.2. Crystal Structure Based Modifications

Nucleic acid aptamers are much smaller than antibodies. In recent years, there are many studies on the crystallization and X-ray diffraction analysis of aptamers or the complex of aptamers and enzyme [90,91,92,93]. It is an effective method to develop modified aptamers with higher affinity and selectivity according to the crystal structures. Autotaxin (ATX) is a plasma lysophospholipase D which can hydrolyze lysophosphatidylcholine (LPC) and generate lysophosphatidic acid (LPA) [94,95]. DNA aptamer RB011 is an inhibitor against ATX. Nureki and coworkers had investigated the crystal structure of ATX in complex with RB011 [96]. The results showed that RB011 inhibited the activity of ATX by preventing its binding to LPC substrates. The hydrophobic pocket of ATX could be occupied by some inhibitors such as HA155 or 3BoA [97,98], but RB011 did not occlude the hydrophobic pocket. Thus, the researchers introduced some hydrophobic groups such as *p*-methyl and *p*-isopropyl into the backbone phosphate of RB011, resulting in RB012 and RB013, respectively. The activities of both RB012 and RB013 (IC_50_ values for LPC were 1.8 and 0.85 nM, respectively) were more potent than that of RB011 (4.4 nM) [96]. These results suggested that modifications aimed to occlude the hydrophobic pocket could significantly increase inhibitory activity. This is a successful modification based on the crystal structural information.

#### 3.3.3. NMR Spectroscopy Guided Aptamer Optimization

Nucleic acid aptamers are widely used for biotechnological or biomedical purpose. High resolution structure information of aptamer–ligand complexes could help reveal the fundamental aspects of nucleic acid folding and nucleic acid-small molecule interactions. Structure information of aptamers and aptamer–ligand complexes constitute the starting point for rational function directed chemical modifications. Duchardt-Ferner E et al. reported the NMR resonance assignment of an RNA aptamer binding to the fluorescent ligand tetramethylrhodamine (TMR) in complex with the ligand 5-carboxy-tetramethylrhodamine (5-TAMRA) as a starting point for a high-resolution structure determination using NMR spectroscopy in solution [99]. This and other reports indicated that NMR guided aptamer optimization could be an optional strategy for aptamer improving binding affinity [100,101,102].

#### 3.3.4. PS2 Walk

**Figure 18:**
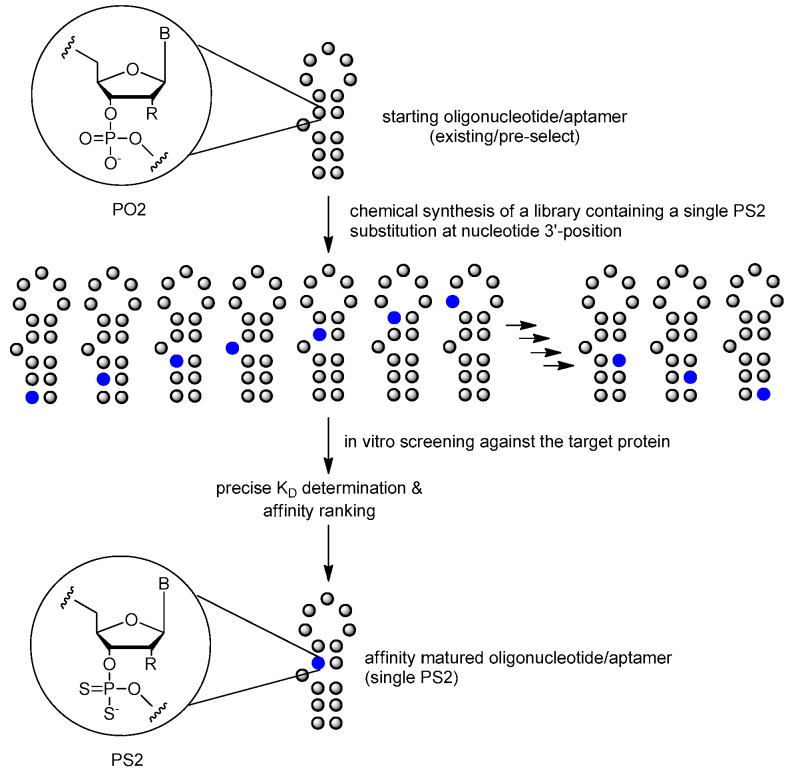
Schematic of the PS2-walk library of sequence variants each containing a single PS2 modification. Modification hot spots along the phosphate backbone of the aptamer could be identified by phosphorodithioate (PS2) substitution on a single nucleotide of nucleic acid sequences.

The binding affinity and specificity of unmodified nucleic acid aptamers are sometimes insufficient for successful implementation as therapeutic agents, compared with monoclonal antibody. Post-SELEX optimization of one Bn-dU and one Nap-dU SOMAmer led to improvements in IL-6 binding (10-fold) and inhibition activity (greater than 20-fold), resulting in lead SOMAmers with sub-nanomolar affinity (*K_d_* = 0.2 nM) and potency (IC_50_ = 0.2 nM) [92]. The PS2 (phosphorodithioate) walk strategy is another option [85,86] (Figure 18). It was reported that the application of the PS2 substitution on a single nucleotide of nucleic acid aptamers could significantly improve target binding affinity by ~1000-fold (from nanomolar to picomolar). An X-ray co-crystal structure of the α-thrombin-PS2-aptamer complex revealed a localized induced-fit folding of the PS2-containing aptamer which leads to increased target interaction [10].

It is worth noting that the effect of PSO substitution (see Section 3.1.4. above) cannot be predicted since the PSO backbone modification is chiral and the chemical synthesis of PSO using phosphoramidite methodology typically results in a mixture of diastereoisomers with a fairly limited influence on the affinity improvement. The promising PS2 derivatives are achiral, representing a class of closely related mimics of natural nucleic acids.

## 4. Conclusions

**Figure 19:**
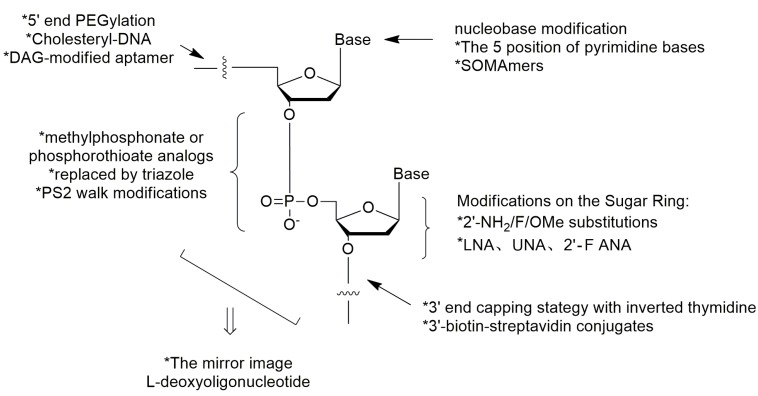
Summary of the chemical modifications of nucleic acid aptamers.

In this review, we introduced the general solid phase synthesis method of nucleic acid aptamers. In addition, a number of chemical modifications of both DNA and RNA aptamers are summarized here. Among all the modifications shown in the Figure 19, 5′-end PEGylation (for resisting renal clearance) and 3′-end capping strategy (for resisting nuclease degradation) with inverted thymidine are the most commonly used strategy in recent studies. These two methods have been used in the aptamers for disease therapy in ongoing or completed clinical trials [15,47].

The nucleobase and phosphodiester linkage modifications (for improving target binding affinity) can also optimize the properties of aptamers. Excitingly, the established technologies provide an opportunity to generate nucleic acid aptamers of substantially improved affinity with a SOMAmer strategy or a single PS2-moiety substitution and without negatively affecting specificity. These technologies also provide crucial insights that could significantly accelerate the development of nucleic acid aptamer-based therapeutics for clinical applications. With the development of post-SELEX modifications of nucleic acid aptamers, the inherent physicochemical characteristics (metabolic instability, insufficient binding affinity and rapid renal filtration) of nucleic acid aptamers have been improved constantly, which provide a strong impetus of developing nucleic acid aptamers for therapeutic purposes (Table 4).

## Figures and Tables

**Table 1:** Chemical modifications of nucleic acid aptamers for resisting nuclease degradation.

Modification Sites	Strategy	Applications
ends of nucleic acid chain	terminal 3′–3′or 5′–5′internucleotide linkage^1^, 3′-biotin conjugates;	[12,15,36,47]
sugar ring of nucleoside	2′-fluoro, 2′-*O*-methyl and 2′-amino-substitutions ^1^, locked nucleic acid (LNA), unlocked nucleic acid (UNA) and 2′-deoxy-2′-fluoro-d-arabinonucleic acid (2′-F ANA);	[25,48,49,52,53,54,55]
phosphodiester linkage	methylphosphonate or phosphorothioate, replaced by triazole;	[23,24,56,57,58,59]
mirror image	l-enantiomeric oligonucleotide aptamers (Spiegelmers)	[66,67,68,69,70]

^1^ 2′-fluoro or 2′-*O*-methyl-substitutions and 3′ end capping with inverted thymidine have been the common strategies in the chemical modifications of nucleic acid aptamers for resisting nuclease degradation.

**Table 2:** Aptamer derivatives for resisting renal clearance.

Modification Sites	Strategy	Applications
ends of nucleic acid chain	5′-end with cholesterol; 5′-end with dialkyl lipids; 5′-end PEGylation ^1^	[32,33,71,72,73,74,75,76,77]

^1^ Terminal PEGylation has been the common strategy in the chemical modifications of nucleic acid aptamers for resisting renal clearance.

**Table 3:** Aptamer derivatives for improving binding affinity and specificity.

Modification Sites	Strategy	Applications
base of nucleoside	5-(*N*-benzylcarboxyamide)-2′-deoxyuridine modification ^1^, Slow Off-rate Modified Aptamers (SOMAmers)	[78,79,80,81,82,83,84]
phosphodiester linkage	phosphorodithioate (PS2) substitution	[10,85,86]

^1^ The benzyl could be replaced by the other functional groups such as naphtyl, triptamino, isobutyl and so on.

**Table 4:** Chemical modifications of nucleic acid aptamers for different purposes.

Strategy	Nuclease Resistance	Improving Binding Affinity and Target Selectivity	Resistance to Renal Clearance
3′-3′inversion/ 3′-T capping	[12,20,21]		
5′-5′inversion	[12]		
3′-biotin conjugates	[20,36]		
2′-fluoro, 2′-*O*-methyl and 2′-amino-substitutions1	[39,48,49]		
locked nucleic acid (LNA)	[52,53]		
unlocked nucleic acid (UNA)	[54,55]		
2′-deoxy-2′-fluoro-d-arabinonucleic acid (2′-F ANA)	[25]		
methylphosphonate	[56]		
phosphorothioate	[23,24]		
replaced by triazole	[57,58,59,60]		
l-enantiomeric oligonucleotide aptamers (Spiegelmers)	[66,67,68,69,70]		
5′-end with cholesterol		[32,33,71]	
5′-end with dialkyl lipids		[72,73]	
5′-end PEGylation		[32,74,75,76,77]	
5-(*N*-benzylcarboxyamide)-2-deoxyuridine modification1, Slow Off-rate Modified Aptamers (SOMAmers)			[78,79,80,81,82,83,84]
phosphorodithioate (PS2) substitution			[10,85,86]
